# Different intensities of physical activity for amyotrophic lateral sclerosis and Parkinson disease: A Mendelian randomization study and meta-analysis

**DOI:** 10.1097/MD.0000000000040141

**Published:** 2024-11-01

**Authors:** Wenyuan Xu, Xianghu Zhao, Jiaying Wang, Yujie Guo, Zhihao Ren, Lian Cai, Shengbing Wu, Meiqi Zhou

**Affiliations:** a Graduate School, Anhui University of Chinese Medicine, Hefei, China; b College of Sports Medicine, Wuhan Sports University, Wuhan, China; c Institute of Acupuncture-Moxibustion and Meridians, Anhui Academy of Traditional Chinese Medicine, Hefei, China; d Anhui Province Key Laboratory of Meridian Viscera Correlationship, Hefei, China.

**Keywords:** amyotrophic lateral sclerosis, Mendelian randomization, Parkinson disease, physical activity

## Abstract

**Background:**

The causal relationships between amyotrophic lateral sclerosis (ALS), Parkinson disease and different intensities of physical activity (PA) are still inconclusive. To evaluate the causal impact of PA on ALS and Parkinson disease (PD), this study integrates evidence from Mendelian randomization (MR) using a meta-analysis approach.

**Methods:**

MR analyses on genetically predicted levels of PA (compose of self-reported moderate-to-vigorous physical activity [MVPA], self-reported vigorous physical activity [VPA], and strenuous sports or other exercises [SSOE]) regarding ALS and PD published up to July 27, 2024, were obtained from PubMed, Scopus, Web of Science, and Embase. De novo MR studies were analyzed utilizing publicly accessible datasets from genome-wide association studies and then meta-analyses were performed to pool the results.

**Results:**

Meta-analyses of results of 12 de novo MR studies analyses and 2 published MR studies indicated that genetic predicted levels of MVPA (odds ratio [OR]: 1.22, 95% confidence interval [CI]: 1.08–1.38), VPA (OR: 1.32, 95% CI: 1.08–1.60), and SSOE (OR: 1.35, 95% CI: 1.07–1.70) were related to a raised risk of ALS, but not causally with PD.

**Conclusion:**

Our findings showed no causal relationships between MVPA, VPA, SSOE, and PD, while MVPA, VPA, and SSOE were associated with increased ALS risk, highlighting the need for targeted PA recommendations for disease management.

## 1. Introduction

**Figure 1.**
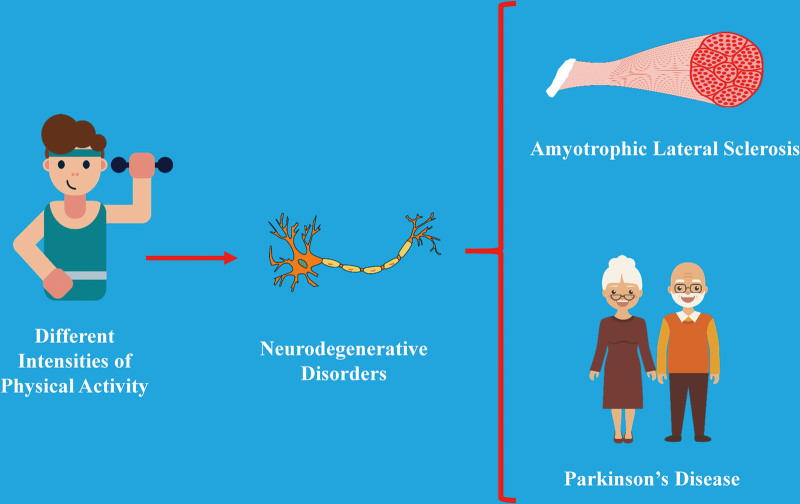
Physical activity and neurodegenerative disorders.

Neurodegenerative disorders, such as amyotrophic lateral sclerosis (ALS) and Parkinson disease (PD), are a significant and increasing public health concern due to their progressive characteristics and impact on motor and cognitive functions.^[1]^ ALS is characterized by the degeneration of motor neurons, leading to muscle weakness and paralysis,^[2]^ while PD is marked by the loss of dopaminergic neurons, resulting in motor symptoms like tremors and rigidity, as well as non-motor issues.^[3]^ The prevalence of ALS and PD are on the rise, largely due to the aging population, imposing substantial burdens on individuals and healthcare systems.^[4]^ Despite the differences in their clinical manifestations, ALS and PD share common pathological mechanisms, including neuronal loss and protein misfolding, which has prompted research into modifiable risk factors, such as physical activity (PA), that may influence their progression (Fig. 1).^[5]^

The relationships between PA and neurodegenerative disorders are complex and have been the subject of numerous studies, yielding inconsistent results. While some researches suggested a protective effect of PA on these conditions, other studies indicated no association or even a potential risk increase.^[6,7]^ This variability may stem from the multifaceted characteristics of PA, which encompasses a broad spectrum of PA with different intensities, durations, and frequencies, as well as the influence of confounding factors such as genetics, lifestyle, and environmental exposures. To address these complexities, Mendelian randomization (MR) is a statistical method used to discern causal relationships between exposure factors and outcomes.^[8]^ Its principle follows the Mendel law of inheritance, which states that genetic variation is distributed randomly during meiosis.^[9]^ MR utilizes genetic variants associated with exposure factors (e.g., PA susceptibility), such as single nucleotide polymorphisms (SNPs), to explore causal relationships with outcome variables.^[10]^ The methodology of MR effectively addresses confounding issues by virtue of the random allocation of genetic alleles during the transmission from parents to offspring.^[11]^ Consequently, these alleles are typically independent of other risk factors. Moreover, since genes are invariant during the progression of disease, MR studies can avoid inverse causality bias.

In recent years, the possible causal relationships between the incidence of ALS, PD, and PA have been explored utilizing MR method.^[12–16]^ We systematically reviewed published studies of MR with the aim of comprehensively evaluating and integrating the evidence regarding the causal association between ALS, PD, and different intensities of PA. To analyze de novo MR, we pooled statistics from the Finnish Genome Study (FinnGen) and other publicly accessible genome-wide association studies (GWAS) datasets. Subsequently, in order to provide new insights for PA intervention in neurodegenerative disorders, published studies and de novo MR analyses were pooled utilizing a meta-analysis approach.

## 2. Methods

### 2.1. Literature retrieval and inclusion criteria

A comprehensive systematic review was performed on the PubMed, Scopus, Web of Science and Embase databases to identify and include relevant MR studies published until July 27, 2024. The search strategy involved the utilization of specific keywords, namely “Mendelian randomization” in combination with “physical activity” or “exercise,” “amyotrophic lateral sclerosis” or “ALS,” and “Parkinson disease” or “PD” to ensure that articles relevant to the research topic were retrieved. Article type is research article. The primary research articles aim to investigate the relationship between ALS, PD and self-reported moderate-to-vigorous physical activity (MVPA), which is defined as self-reported engagement in any physical activity that requires a sustained moderate effort for at least 10 minutes and noticeably increases heart rate and breathing.^[17]^ Additionally, the studies examine the relationship between ALS, PD and self-reported vigorous physical activity (VPA), which is defined as the number of days engaging in high-intensity physical activity for at least 10 minutes or more in a typical week.^[17]^ Lastly, the studies analyze the relationship between ALS, PD and strenuous sports or other exercises (SSOE), which is defined as engaging in intense physical activities or other forms of exercise for 2 to 3 days or more within the past 4 weeks, with each session lasting 15 to 30 minutes or longer.^[17]^ When multiple publications derived from GWAS with the identical outcome (same participants) and the different number of instrumental variables resulted in different odds ratio (OR) estimates for the outcome, they were included together for meta-analysis. No limitation derived from sample size was imposed. We excluded primary literatures with exposure factor units that did not in line with this study. The study was conducted in accordance with STROBE-MR checklist. This review was performed without registration, and protocol is unavailable.

### 2.2. Data extraction

Data for each study were extracted including: last name and year of publication of the first author; number of instrumental variables for PA exposure factors and the consortium or study from which they were derived; OR estimates and 95% confidence intervals (CIs) for the diseases; and results of inverse variance weighting and sensitivity analyses (including MR-Egger and weighted median methods). Additional extracted information included the sample size, comprising the number of cases and noncases. Data extraction was conducted by 2 investigators and independently confirmed by other investigators.

### 2.3. De novo MR analyses

In cases where MR findings from this study were unavailable, de novo MR analyses were integrated utilizing publicly accessible datasets from the FinnGen study, and other GWAS meta-analyses or consortiums. The data utilized in our study was sourced from the IEU OpenGWAS project,^[18]^ primarily consisting of publicly accessible datasets, specifically focusing on a sample population with mixed-sex individuals of European ancestry. Information regarding diseases was obtained from comprehensive nationwide registries. Further information on quality control, methods, and genotype data is available in IEU OpenGWAS project under GWAS ID (Table S1, Supplemental Digital Content, http://links.lww.com/MD/N826). All de novo MR analyses were conducted utilizing R software (version 4.2.3) with “TwoSampleMR” package.

De novo MR analyses were performed for ALS and PD, and integration of statistical data for these results were obtained from publicly accessible datasets. Instrumental variables in each de novo MR analysis were composed of independent genetic variants associated with MVPA, VPA, or SSOE that were genome-wide significant at the level of significance (*P* < 5 × 10^‐8^).^[17]^ The criterion for selection of instrumental variables was an insignificant linkage disequilibrium (marked as R^2^ < 0.01) and *F*-statistic > 10 (Table S2, Supplemental Digital Content, http://links.lww.com/MD/N826). All do novo Mendelian randomizations are calculated for statistical power using the online tool available at: https://shiny.cnsgenomics.com/mRnd/.

All instrumental variables for exposure factors in the de novo MR analyses were derived from the UK Biobank data and 19, 7, and 14 SNPs were screened and analyzed as instrumental variables for MVPA, VPA, and SSOE, separately (Table S2, Supplemental Digital Content, http://links.lww.com/MD/N826). The primary analysis adopted the inverse-variance weighted method, which follows a multiplicative random-effects model.^[19]^ Sensitivity analyses were performed utilizing the MR-Egger and weighted median methods.

### 2.4. Meta-analysis

When multiple MR estimates were obtainable for the identical result on the basis of distinct samples, a meta-analysis was performed utilizing the metan command in Stata to obtain a combined estimate.^[20]^ Figures were generated using R software, version 4.3.1. Every reported estimate was shown per standard deviation raise in genetic susceptibility to PA (intensity of participation in PA) index. Sensitivity analyses were performed utilizing estimates based on the MR-Egger and weighted median methods. Additionally, sensitivity analyses involved the exclusion of consortia comprising individuals of non-European and non-mixed-sex ancestries. Since the instrumental variables for exposure factors and outcomes in Mendelian randomization studies are SNPs rather than patients themselves, the quality of the literature was not assessed according to the PRISMA guidelines. However, each of the included Mendelian randomization studies met the 3 fundamental assumptions. All Mendelian randomizations included in the meta-analysis were also calculated for statistical power.

### 2.5. Role of the funding source

The funders did not participate in the study design; data collection, analysis, and interpretation; report writing; and in the determination to submit the manuscript for publication, or any aspect pertinent to the study.

### 2.6. Ethics statement

All studies comprised in the meta-analyses were reviewed and approved by the ethic committee, and patients/participants presented explicit and informed consent to participate in this study.

## 3. Results

### 3.1. Literature retrieval, study inclusion, and de novo MR analyses

A retrieval of the PubMed, Scopus, Web of Science and Embase databases yielded 28 relevant articles, leaving 22 after removal of duplicates. After excluding studies that could not satisfy the criteria for the type of exposure factors in our study, 2 articles were identified for inclusion in this meta-analysis, one of which explored the relationship between PA and ALS, and the other explored the relationship between PA and PD.^[12,14]^ In addition, we conducted 12 de novo Mendelian randomization studies. The statistical power of all Mendelian randomization studies is >80% (Tables S3–S5, Supplemental Digital Content, http://links.lww.com/MD/N826). Figure S1, Supplemental Digital Content, http://links.lww.com/MD/N825 shows the selection and design of this study.

### 3.2. Study description

All included studies investigating PA utilized genetic variants identified from a GWAS involving a population of 377,000 individuals with European ancestry.^[21]^ Outcome data for MR analyses were all obtained from large publicly available GWAS meta-analyses (consortium), FinnGen studies, or other data sources. All of the studies in this paper mainly used the inverse variance weighting (IVW) method to derive the results of PA, and the weighted median and the MR-Egger method to corroborate the results of the IVW method. The results of the IVW method for the effect of exposure factors of 4 different intensities of PA on ALS and PD can be found in Table S3, Supplemental Digital Content, http://links.lww.com/MD/N826 (MVPA), Table S4, Supplemental Digital Content, http://links.lww.com/MD/N826 (VPA), and Table S5, Supplemental Digital Content, http://links.lww.com/MD/N826 (SSOE). The results of the weighted median and the MR-Egger method for the effect of exposure factors of 4 different intensities of PA on ALS and PD can be found in Table S6 (MVPA), Table S7, Supplemental Digital Content, http://links.lww.com/MD/N826 (VPA), and Table S8, Supplemental Digital Content, http://links.lww.com/MD/N826 (SSOE).

### 3.3. Causal relationship between ALS, PD, and MVPA

**Figure 2.**
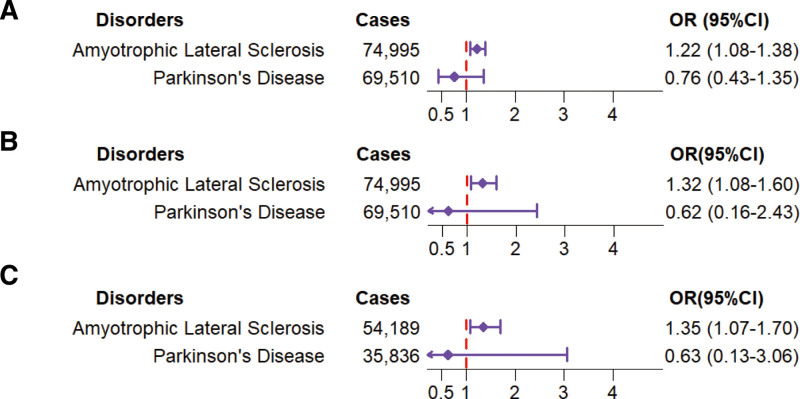
Results of meta-analysis of OR and 95% CI for the association of MVPA, VPA, and SSOE with risk of ALS and PD: (A) MVPA; (B) VPA; (C) SSOE. OR = odds ratio, CI = confidence interval, MVPA = self-reported moderate-to-vigorous physical activity, VPA = self-reported vigorous physical activity, SSOE = strenuous sports or other exercises, ALS = amyotrophic lateral sclerosis, PD = Parkinson disease.

Genetic susceptibility to MVPA was found to be related to a raised risk of ALS (OR: 1.22, 95% CI: 1.08–1.38). However, no causal association was observed between MVPA and PD (OR: 0.76, 95% CI: 0.43–1.35) (Fig. 2A; Table S3, Supplemental Digital Content, http://links.lww.com/MD/N826).

### 3.4. Causal relationship between ALS, PD and VPA

Genetic susceptibility to VPA was found to be related to a raised risk of ALS (OR: 1.32, 95% CI: 1.08–1.60). However, no causal association was observed between VPA and PD (OR: 0.62, 95% CI: 0.16–2.43) (Fig. 2B; Table S4, Supplemental Digital Content, http://links.lww.com/MD/N826).

### 3.5. Causal relationship between ALS, PD and SSOE

Genetic susceptibility to SSOE was found to be related to a raised risk of ALS (OR: 1.35, 95% CI: 1.07–1.70). However, no causal association was observed between SSOE and PD (OR: 0.63, 95% CI: 0.13–3.06) (Fig. 2C; Table S5, Supplemental Digital Content, http://links.lww.com/MD/N826).

### 3.6. Sensitivity analyses

The results obtained through the MR-Egger and weighted median methods generally in line with the primary conclusions, although the precision of these results was low to remarkably uncertain in part of MR analyses. Additionally, there was insufficient evidence for some MR-Egger tests (*P* < .05), suggesting possible horizontal pleiotropy (Tables S6–S8, Supplemental Digital Content, http://links.lww.com/MD/N826).

## 4. Discussion

Our study used a MR approach coupled with meta-analytic techniques to indicate the complex relationships between different intensities of PA and the risk of neurodegenerative disorders, with a specific focus on ALS and PD. Our findings, which reported a potential increase in ALS risk associated with MVPA, VPA, and SSOE, contrast with the traditional view that PA is universally protective against neurodegenerative disorders.^[5,22,23]^ However, the lack of a significant association between PA and PD risk. These differences require a deeper exploration of the underlying mechanisms and a reevaluation of the current recommendations for PA in the background of neurodegenerative disorders.

Previous researches have reported that PA may worsen the progression of ALS, which is consistent with our findings. In our work, we utilized MR to genetically investigate the impact of MVPA, VPA, and SSOE on the risk of ALS. We found that MVPA, VPA, and SSOE increased the risk of ALS, with the underlying biological mechanism potentially being that high-intensity PA lead to elevated oxidative stress, exacerbating the characteristic motor neuron degeneration in ALS and reducing synaptic plasticity, thereby slowing down neuroprotective and reparative mechanisms.^[6,24–26]^ Additionally, the increased metabolic demands of motor neurons during PA may accelerate the pathological processes in genetically susceptible individuals.^[27]^ Despite support from these hypotheses and theories, there is currently a lack of sufficient scientific research to clearly indicate the biological mechanisms by which PA intervenes in ALS. The impact of PA on ALS and its potential therapeutic effects need further investigation.

The lack of a significant association between PA and PD risk in our study. It suggested that the relationship between PA and PD may be more complex and potentially moderated by factors such as disease stage, genetic predisposition, and the specific motor and non-motor symptoms experienced by individuals with PD.^[28–31]^ Furthermore, it is possible that the neuroprotective effects of PA in PD are more pronounced in later stages of the disease, where the pathological processes are more advanced, and the benefits of PA might be more obvious.^[32–34]^

The biological mechanisms through which different intensities of PA may influence the risk of neurodegenerative disorders require further study. Future researches should explore the role of molecular pathways, such as inflammation, autophagy, and mitochondrial function, which may be differentially affected by varying levels of PA.^[35,36]^ Moreover, the potential for gene–environment interactions, where genetic susceptibility modifies the effect of PA on disease risk, should be examined in future studies.^[37,38]^

To our knowledge, this is the first MR study and meta-analysis that provides new insight into the causal associations between ALS, PD and different intensities of PA. One notable strength of our study is MR can exclude biases in observational studies, including confounding factors and inverse causality. This is achieved by using genetic variants as instrumental variables in place of interested exposure (e.g., exercise), which are usually relatively stable and not affected by other factors. However, MR analysis is based on 3 core assumptions. (I) SNPs are strongly associated with PA. The SNPs selected for this study were significantly associated with genetic susceptibility to PA and the instrumental strength met the criteria, defined as *F*-statistic > 10 (Table S2, Supplemental Digital Content, http://links.lww.com/MD/N826); (II) SNPs are independent of confounders; (III) SNPs must only affect multiple diseases via PA. While it is impossible to completely exclude the potential influence of pleiotropy on the findings, most studies reported results using robust methods such as the MR-Egger and weighted median approaches, which are less susceptible to pleiotropic bias, and reliable findings were observed in these secondary analyses. It is of importance to interpret results from MR-Egger analyses with caution, as some estimates exhibited low to highly imprecise, as evidenced by wide 95% CIs.

The possible competing risk of bias in this study, which could have affected some of the findings, was also present in the MR analyses of adverse exposures and late-onset disease. Advancing age at study enrollment usually causes an increase in this bias.^[39]^ Selection bias may have a less influence on MR findings than other biases.^[40]^ The findings of MR studies can be affected by selection bias if collisions between genetic variants and confounders related to the exposure–outcome relationship affect study inclusion.^[40]^ Population stratification bias is another potential bias in MR studies. For this study, the majority of the included studies comprised of individuals of European ancestry exclusively, and genetic principal components adjusted for other remaining stratification bias. Finally, the possible overlap in sample size may also cause potential bias.

Our findings also have implications for clinical practice and public health guidelines. The potential increase in ALS risk associated with MVPA, VPA, and SSOE suggested that a single solution to PA recommendations may not be appropriate for all individuals, particularly those with a genetic predisposition to neurodegenerative disorders. Therefore, a personalized PA program must be carried out, taking into account an individual’s disease stage and personal preferences, to optimize health outcomes.

## 5. Conclusion

In summary, we are pleased to demonstrate through the integration of extensive genetic evidence that MVPA, VPA, and SSOE can lead to an increased risk of ALS, without causal relationship for PD. Therefore, the intensity of different intensities PA is crucial for the progression of ALS, PD, and the rational and appropriate prescription of PA is an essential component of positive health promotion in public health. In the future, we may explore the specific mechanisms by which PA affects ALS and PD, including metabolic and inflammatory pathways, thereby providing new insights for disease prevention and treatment.

## Author contributions

**Conceptualization:** Shengbing Wu, Meiqi Zhou.

**Data curation:** Wenyuan Xu, Xianghu Zhao, Jiaying Wang, Zhihao Ren.

**Formal analysis:** Xianghu Zhao.

**Methodology:** Xianghu Zhao, Yujie Guo, Zhihao Ren, Lian Cai.

**Software:** Wenyuan Xu, Xianghu Zhao, Yujie Guo.

**Writing – original draft:** Wenyuan Xu.

**Writing – review & editing:** Wenyuan Xu.

## Supplementary Material


