# Supplementary material for: Different intensities of physical activity for amyotrophic lateral sclerosis and Parkinson disease: A Mendelian randomization study and meta-analysis
Source: Medicine (Baltimore). 2024 Nov 1;103(44):e40141. doi: 10.1097/MD.0000000000040141 (PMC11537586; doi:10.1097/MD.0000000000040141)
Supplement: Supplementary file 1 [file medi-103-e40141-s001.docx]

**Table S1.** Instrumental variables information extracted from IEU OpenGWAS project.

| **Disorders** | **GWAS ID** | **Data source** |
| --- | --- | --- |
| ALS | ieu-a-1085  ebi-a-GCST005647 | Project MinE  NA |
| PD | ieu-b-7  finn-b-G6_PARKINSON | IPDGC  FinnGen |

ALS, amyotrophic lateral sclerosis; PD, Parkinson’s disease; IPDGC, international parkinson's disease genomics consortium; NA, not available.

**Table S2.** Single-nucleotide polymorphisms used as instrumental variables in the de novo Mendelian randomization analyses of UK Biobank data.

| **Phenotype** | **Comparison or units** | **SNP** | **Chr** | **EA** | **OA** | **Beta** | **SE** | ***P* value** | **F-statistic** |
| --- | --- | --- | --- | --- | --- | --- | --- | --- | --- |
| MVPA | 1-SD in MET-minutes/week after inverse normalization | rs2942127 | 1 | A | G | -0.0160 | 0.0029 | 3.30E-08 | 31 |
| MVPA |  | rs1974771 | 2 | A | G | 0.0213 | 0.0037 | 6.60E-09 | 34 |
| MVPA |  | rs2114286 | 3 | G | A | 0.0122 | 0.0022 | 3.30E-08 | 31 |
| MVPA |  | rs877483 | 3 | C | T | -0.0122 | 0.0022 | 4.00E-08 | 30 |
| MVPA |  | rs2035562 | 3 | G | A | 0.0139 | 0.0024 | 3.90E-09 | 35 |
| MVPA |  | rs1972763 | 4 | T | C | -0.0128 | 0.0023 | 3.30E-08 | 31 |
| MVPA |  | rs77742115 | 5 | C | T | 0.0183 | 0.0032 | 9.60E-09 | 33 |
| MVPA |  | rs2854277 | 6 | T | C | -0.0320 | 0.0051 | 2.60E-10 | 40 |
| MVPA |  | rs7804463 | 7 | C | T | -0.0150 | 0.0022 | 1.20E-11 | 46 |
| MVPA |  | rs921915 | 7 | C | T | 0.0139 | 0.0022 | 5.70E-10 | 38 |
| MVPA |  | rs1186721 | 7 | A | G | 0.0130 | 0.0024 | 4.40E-08 | 30 |
| MVPA |  | rs1043595 | 7 | A | G | -0.0144 | 0.0025 | 4.30E-09 | 34 |
| MVPA |  | rs2988004 | 9 | G | T | 0.0132 | 0.0022 | 4.10E-09 | 35 |
| MVPA |  | rs7326482 | 13 | T | G | 0.0130 | 0.0023 | 1.60E-08 | 32 |
| MVPA |  | rs10145335 | 14 | A | G | 0.0141 | 0.0025 | 2.70E-08 | 31 |
| MVPA |  | rs12912808 | 15 | T | C | -0.0175 | 0.0031 | 1.70E-08 | 32 |
| MVPA |  | rs4886868 | 15 | G | T | 0.0125 | 0.0023 | 3.50E-08 | 30 |
| MVPA |  | rs429358 | 19 | C | T | 0.0220 | 0.0031 | 6.10E-13 | 52 |
| MVPA |  | rs1921981 | 21 | A | G | -0.0130 | 0.0024 | 3.80E-08 | 30 |
| VPA | ≥3 vs 0 days/week | rs6667222 | 1 | C | A | -0.0087 | 0.0015 | 8.70E-09 | 33 |
| VPA |  | rs1248860 | 3 | A | G | 0.0098 | 0.0013 | 1.10E-13 | 55 |
| VPA |  | rs9276758 | 6 | A | G | -0.0080 | 0.0014 | 1.40E-08 | 32 |
| VPA |  | rs2764261 | 6 | G | A | -0.0091 | 0.0014 | 2.00E-11 | 45 |
| VPA |  | rs13243553 | 7 | A | G | -0.0087 | 0.0013 | 9.00E-11 | 42 |
| VPA |  | rs328902 | 7 | T | C | 0.0088 | 0.0014 | 5.50E-10 | 38 |
| VPA |  | rs3781411 | 10 | T | C | -0.0126 | 0.0020 | 3.00E-10 | 40 |
| SSOE | ≥ 2-3 vs 0 for days per week | rs1200154 | 1 | A | G | 0.0063 | 0.0011 | 3.90E-08 | 30 |
| SSOE |  | rs2994326 | 1 | C | T | 0.0080 | 0.0015 | 4.50E-08 | 30 |
| SSOE |  | rs288070 | 2 | A | G | 0.0106 | 0.0019 | 1.90E-08 | 32 |
| SSOE |  | rs62253088 | 3 | C | T | -0.0109 | 0.0012 | 1.00E-19 | 83 |
| SSOE |  | rs7627864 | 3 | G | C | -0.0066 | 0.0011 | 7.60E-09 | 33 |
| SSOE |  | rs4865667 | 5 | T | C | -0.0066 | 0.0012 | 1.00E-08 | 33 |
| SSOE |  | rs159544 | 5 | G | A | 0.0070 | 0.0012 | 1.30E-09 | 37 |
| SSOE |  | rs10946808 | 6 | G | A | 0.0077 | 0.0013 | 9.90E-10 | 37 |
| SSOE |  | rs1265178 | 6 | A | G | -0.0073 | 0.0013 | 3.20E-08 | 31 |
| SSOE |  | rs896302 | 6 | T | C | -0.0070 | 0.0012 | 1.70E-08 | 32 |
| SSOE |  | rs4411372 | 13 | C | T | 0.0070 | 0.0013 | 2.00E-08 | 31 |
| SSOE |  | rs75930676 | 14 | C | T | 0.0158 | 0.0026 | 2.00E-09 | 36 |
| SSOE |  | rs166840 | 17 | A | G | -0.0076 | 0.0012 | 3.10E-11 | 44 |
| SSOE |  | rs111901094 | 19 | T | G | -0.0088 | 0.0015 | 3.00E-09 | 35 |

MVPA, self-reported moderate-to-vigorous physical activity; VPA, self-reported vigorous physical activity; SSOE, strenuous sports or other exercises; MET, metabolic equivalent of task; SD, standard deviation; EA, effect allele; OA, other allele; SE, standard error.

**Table S3.** Mendelian randomization studies included in the meta-analyses of genetic liability to **self-reported moderate-to-vigorous physical activity** in relation to amyotrophic lateral sclerosis and parkinson’s disease

| **Disorders** | **Study** | **Cases** | **Noncases** | **SNPs** | **OR** | **LB** | **UB** | **Power** | **First author, year** |
| --- | --- | --- | --- | --- | --- | --- | --- | --- | --- |
| ALS | Project MinE | 12,577 | 23,475 | 18 | 1.09 | 0.95 | 1.26 | 93.6% | De novo MR analysis, 2023 |
| ALS | NA | 20,806 | 59,804 | 74 | 1.86 | 1.40 | 2.47 | 86.3% | De novo MR analysis, 2023 |
| ALS | GWAS meta-analysis | 20,806 | 59,804 | 7 | 2.07 | 0.73 | 5.88 | 88.8% | Zhang et al, 2021 |
| ALS | GWAS meta-analysis | 20,806 | 59,804 | 5 | 0.86 | 0.12 | 6.37 | 82.2% | Liao et al, 2022 |
| **ALS** | **Meta-analysis** | **74,995** | **202,887** |  | **1.22** | **1.08** | **1.38** |  |  |
| PD | FinnGen | 2,162 | 216,630 | 18 | 1.10 | 0.33 | 3.67 | 80.6% | De novo MR analysis, 2023 |
| PD | GWAS meta-analysis | 33,674 | 449,056 | 7 | 0.75 | 0.20 | 2.754 | 94.5% | Liao et al, 2022 |
| PD | IPDGC | 33,674 | 449,056 | 18 | 0.67 | 0.32 | 1.40 | 91.2% | De novo MR analysis, 2023 |
| **PD** | **Meta-analysis** | **69,510** | **1,114,742** |  | **0.76** | **0.43** | **1.35** |  |  |

ALS, amyotrophic lateral sclerosis; PD, parkinson’s disease; IPDGC, international parkinson's disease genomics consortium; OR, odds ratio; LB, lower bound of the 95% confidence interval; UB, upper bound of the 95% confidence interval.

**Table S4.** Mendelian randomization studies included in the meta-analyses of genetic liability to **self-reported vigorous physical activity** in relation to amyotrophic lateral sclerosis and parkinson’s disease.

| **Disease** | **Study** | **Cases** | **Noncases** | **SNPs** | **OR** | **LB** | **UB** | **Power** | **First author, year** |
| --- | --- | --- | --- | --- | --- | --- | --- | --- | --- |
| ALS | Project MinE | 12,577 | 23,475 | 7 | 1.23 | 0.91 | 1.66 | 91.4% | De novo MR analysis, 2023 |
| ALS | NA | 20,806 | 59,804 | 30 | 2.30 | 1.28 | 4.13 | 87.6% | De novo MR analysis, 2023 |
| ALS | GWAS meta-analysis | 20,806 | 59,804 | 5 | 1.22 | 0.90 | 1.65 | 85.6% | Zhang et al, 2021 |
| ALS | GWAS meta-analysis | 20,806 | 59,804 | 6 | 1.25 | 0.51 | 3.08 | 82.3% | Liao et al, 2022 |
| **ALS** | **Meta-analysis** | **74,995** | **202,887** |  | **1.32** | **1.08** | **1.60** |  |  |
| PD | FinnGen | 2,162 | 216,630 | 7 | 0.64 | 0.01 | 2.88E+01 | 82.7% | De novo MR analysis, 2023 |
| PD | GWAS meta-analysis | 33,674 | 449,056 | 5 | 0.83 | 0.08 | 8.529 | 87.7% | Liao et al, 2022 |
| PD | IPDGC | 33,674 | 449,056 | 7 | 0.50 | 0.08 | 3.35 | 89.4% | De novo MR analysis, 2023 |
| **PD** | **Meta-analysis** | **69,510** | **1,114,742** |  | **0.62** | **0.16** | **2.43** |  |  |

ALS, amyotrophic lateral sclerosis; PD, parkinson’s disease; IPDGC, international parkinson's disease genomics consortium; OR, odds ratio; LB, lower bound of the 95% confidence interval; UB, upper bound of the 95% confidence interval.

**Table S5.** Mendelian randomization studies included in the meta-analyses of genetic liability to **strenuous sports or other exercises** in relation to amyotrophic lateral sclerosis and parkinson’s disease.

| **Disease** | **Study** | **Cases** | **Noncases** | **SNPs** | **OR** | **LB** | **UB** | **Power** | **First author, year** |
| --- | --- | --- | --- | --- | --- | --- | --- | --- | --- |
| ALS | Project MinE | 12,577 | 23,475 | 10 | 1.32 | 0.94 | 1.87 | 86.7% | De novo MR analysis, 2023 |
| ALS | NA | 20,806 | 59,804 | 30 | 2.30 | 1.28 | 4.13 | 90.2% | De novo MR analysis, 2023 |
| ALS | GWAS meta-analysis | 20,806 | 59,804 | 6 | 1.13 | 0.79 | 1.63 | 92.8% | Zhang et al, 2021 |
| **ALS** | **Meta-analysis** | **54,189** | **143,083** |  | **1.35** | **1.07** | **1.70** |  |  |
| PD | FinnGen | 2,162 | 216,630 | 12 | 1.04 | 0.06 | 1.86E+01 | 83.5% | De novo MR analysis, 2023 |
| PD | IPDGC | 33,674 | 449,056 | 7 | 0.50 | 0.08 | 3.35 | 87.7% | De novo MR analysis, 2023 |
| **PD** | **Meta-analysis** | **35,836** | **665,686** |  | **0.63** | **0.13** | **3.06** |  |  |

ALS, amyotrophic lateral sclerosis; PD, Parkinson’s disease; IPDGC, international parkinson's disease genomics consortium; OR, odds ratio; LB, lower bound of the 95% confidence interval; UB, upper bound of the 95% confidence interval.

**Table S6.** Sensitivity analysis results for genetic liability to **self-reported moderate-to-vigorous physical activity** and studied outcomes based on the weighted median and MR-Egger methods.

| **Disease** | **Study** | **Cases** | **Noncases** | **SNPs** | **Weighted median** | | |  | **MR- Egger** | | | ***P-*value for MR-Egger intercept*** | **First author, year** |
| --- | --- | --- | --- | --- | --- | --- | --- | --- | --- | --- | --- | --- | --- |
| **NERVOUS SYSTEM** |  |  |  |  | **OR** | **LB** | **UB** |  | **OR** | **LB** | **UB** |  |  |
| ALS | Project MinE | 12,577 | 23,475 | 18 | 1.25 | 0.36 | 4.33 |  | 0.48 | 0.00 | 1.14E+03 | 0.7260 | De novo MR analysis, 2023 |
| ALS | NA | 20,806 | 59,804 | 74 | 1.94 | 0.71 | 5.30 |  | 3.43 | 0.22 | 5.40E+01 | 0.8260 | De novo MR analysis, 2023 |
| ALS | GWAS meta-analysis | 20,806 | 59,804 | 7 | 1.05 | 0.88 | 1.24 |  | 1.52 | 0.65 | 3.52 | 0.4545 | Zhang et al, 2021 |
| ALS | GWAS meta-analysis | 20,806 | 59,804 | 5 | 1.49 | 1.04 | 2.14 |  | 2.74 | 0.79 | 9.45 | 0.5295 | Liao et al, 2022 |
| **ALS** | **Meta-analysis** | **74,995** | **202,887** |  | **1.13** | **0.97** | **1.32** |  | **1.88** | **0.96** | **3.69** |  |  |
| PD | FinnGen | 2,162 | 216,630 | 18 | 1.26 | 0.25 | 6.38 |  | 3.55E+03 | 6.75 | 1.87E+06 | 0.0206 | De novo MR analysis, 2023 |
| PD | GWAS meta-analysis | 33,674 | 449,056 | 7 | 0.42 | 0.10 | 1.80 |  | 0.24 | 0.00 | 5.88E+02 | 0.7830 | Liao et al, 2022 |
| PD | IPDGC | 33,674 | 449,056 | 18 | 0.45 | 0.17 | 1.21 |  | 0.30 | 0.00 | 1.78E+01 | 0.6966 | De novo MR analysis, 2023 |
| **PD** | **Meta-analysis** | **69,510** | **1,114,742** |  | **0.55** | **1.26** | **1.13** |  | **3.02** | **0.13** | **6.97E+01** |  |  |

ALS, amyotrophic lateral sclerosis; PD, parkinson’s disease; IPDGC, international parkinson's disease genomics consortium; OR, odds ratio; LB, lower bound of the 95% confidence interval; UB, upper bound of the 95% confidence interval.

*A *P* value <0.05 provides evidence of possible pleiotropy.

**Table S7.** Sensitivity analysis results for genetic liability to **self-reported vigorous physical activity** and studied outcomes based on the weighted median and MR-Egger methods.

| **Disease** | **Study** | **Cases** | **Noncases** | **SNPs** | **Weighted median** | | |  | **MR- Egger** | | | ***P-*value for MR-Egger intercept*** | **First author, year** |
| --- | --- | --- | --- | --- | --- | --- | --- | --- | --- | --- | --- | --- | --- |
| **NERVOUS SYSTEM** |  |  |  |  | **OR** | **LB** | **UB** |  | **OR** | **LB** | **UB** |  |  |
| ALS | Project MinE | 12,577 | 23,475 | 7 | 1.19 | 0.82 | 1.73 |  | 0.89 | 0.21 | 3.84 | 0.6920 | De novo MR analysis, 2023 |
| ALS | NA | 20,806 | 59,804 | 30 | 2.28 | 0.42 | 1.23E+01 |  | 3.32 | 0.00 | 1.46E+05 | 0.9650 | De novo MR analysis, 2023 |
| ALS | GWAS meta-analysis | 20,806 | 59,804 | 5 | 1.23 | 0.81 | 1.86 |  | 1.56 | 0.10 | 2.45E+01 | 0.8727 | Zhang et al, 2021 |
| ALS | GWAS meta-analysis | 20,806 | 59,804 | 6 | 2.40 | 1.13 | 5.09 |  | 9.24 | 0.06 | 1.36E+03 | 0.5872 | Liao et al, 2022 |
| **ALS** | **Meta-analysis** | **74,995** | **202,887** |  | **1.33** | **1.03** | **1.72** |  | **1.17** | **0.34** | **4.03** |  |  |
| PD | FinnGen | 2,162 | 216,630 | 7 | 4.94 | 0.09 | 2.73E+02 |  | 1.33E+04 | 0.00 | 4.19E+17 | 0.5549 | De novo MR analysis, 2023 |
| PD | GWAS meta-analysis | 33,674 | 449,056 | 5 | 0.98 | 0.09 | 1.05E+01 |  | 0.01 | 0.00 | 2.45E+06 | 0.6590 | Liao et al, 2022 |
| PD | IPDGC | 33,674 | 449,056 | 7 | 0.30 | 0.04 | 2.50 |  | 1.15 | 0.00 | 1.66E+07 | 0.9247 | De novo MR analysis, 2023 |
| **PD** | **Meta-analysis** | **69,510** | **1,114,742** |  | **0.69** | **0.16** | **3.00** |  | **0.11** | **0.00** | **1.18E+03** |  |  |

ALS, amyotrophic lateral sclerosis; PD, parkinson’s disease; IPDGC, international parkinson's disease genomics consortium; OR, odds ratio; LB, lower bound of the 95% confidence interval; UB, upper bound of the 95% confidence interval.

*A *P* value <0.05 provides evidence of possible pleiotropy.

**Table S8.** Sensitivity analysis results for genetic liability to **strenuous sports or other exercises** and studied outcomes based on the weighted median and MR-Egger methods.

| **Disease** | **Study** | **Cases** | **Noncases** | **SNPs** | **Weighted median** | | |  | **MR- Egger** | | | ***P-*value for MR-Egger intercept*** | **First author, year** |
| --- | --- | --- | --- | --- | --- | --- | --- | --- | --- | --- | --- | --- | --- |
| **NERVOUS SYSTEM** |  |  |  |  | **OR** | **LB** | **UB** |  | **OR** | **LB** | **UB** |  |  |
| ALS | Project MinE | 12,577 | 23,475 | 10 | 1.23 | 0.77 | 1.97 |  | 1.04 | 0.26 | 4.14 | 0.9000 | De novo MR analysis, 2023 |
| ALS | NA | 20,806 | 59,804 | 30 | 1.18 | 0.77 | 1.81 |  | 0.34 | 0.06 | 1.94 | 0.1584 | De novo MR analysis, 2023 |
| ALS | GWAS meta-analysis | 20,806 | 59,804 | 6 | 2.40 | 1.13 | 5.09 |  | 9.24 | 0.06 | 1.36E+03 | 0.5872 | Zhang et al, 2021 |
| **ALS** | **Meta-analysis** | **54,189** | **143,083** |  | **1.33** | **1.00** | **1.79** |  | **0.76** | **0.26** | **2.19** |  |  |
| PD | FinnGen | 2,162 | 216,630 | 12 | 0.30 | 0.04 | 2.50 |  | 1.15 | 0.00 | 1.66E+07 | 0.9247 | De novo MR analysis, 2023 |
| PD | IPDGC | 33,674 | 449,056 | 7 | 1.53 | 0.03 | 6.94E+01 |  | 5.95 | 0.00 | 9990690.00 | 0.8127 | De novo MR analysis, 2023 |
| **PD** | **Meta-analysis** | **35,836** | **665,686** |  | **0.44** | **0.07** | **2.81** |  | **2.93** | **0.00** | **1.46E+05** |  |  |

ALS, amyotrophic lateral sclerosis; PD, parkinson’s disease; IPDGC, International Parkinson's Disease Genomics Consortium; OR, odds ratio; LB, lower bound of the 95% confidence interval; UB, upper bound of the 95% confidence interval.

*A *P* value <0.05 provides evidence of possible pleiotropy.
