# Supplementary material for: Different intensities of physical activity for amyotrophic lateral sclerosis and Parkinson disease: A Mendelian randomization study and meta-analysis
Source: Medicine (Baltimore). 2024 Nov 1;103(44):e40141. doi: 10.1097/MD.0000000000040141 (PMC11537586; doi:10.1097/MD.0000000000040141)
Supplement: Supplementary file 2 [file medi-103-e40141-s002.docx]

**Figure S1.** Systematic review and study selection


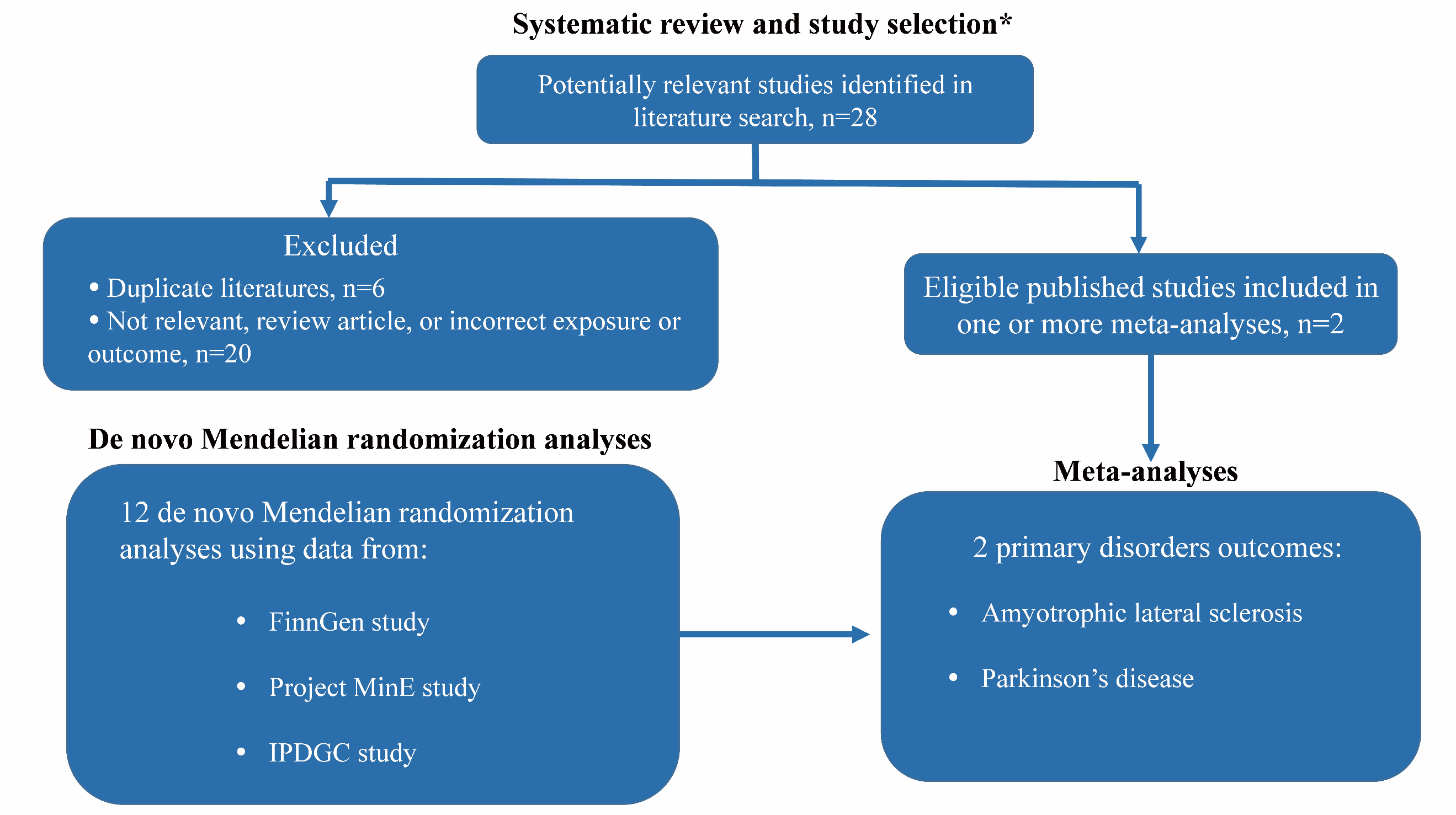


*Inclusion criteria: Original full-text article that presents results for the associations of genetic liability to self-reported moderate-to-vigorous physical activity, self-reported vigorous physical activity or strenuous sports or other exercises with risk of amyotrophic lateral sclerosis, parkinson's disease or schizophrenia. Exclusion criteria: Duplicate literatures based on the same or overlapping study sample, and studies that did not include one of the three exposure factors of our study.
